# Validity of the microsoft kinect system in assessment of compensatory stepping behavior during standing and treadmill walking

**DOI:** 10.1186/s11556-017-0172-8

**Published:** 2017-03-07

**Authors:** Guy Shani, Amir Shapiro, Goldstein Oded, Kagan Dima, Itshak Melzer

**Affiliations:** 1 Department of Software and Information Systems Engineering, Faculty of Engineering Sciences, Ben-Gurion University, Beer-Sheva, Israel; 2 Department of Mechanical Engineering, Faculty of Engineering Sciences, Ben-Gurion University, Beer-Sheva, Israel; 3 Schwartz Movement Analysis & Rehabilitation Laboratory, Physical Therapy Department, Recanati School for Community Health Professions, Faculty of Health Sciences, Ben-Gurion University of the Negev, P.O.B. 653, Beer-Sheva, 84105 Israel

**Keywords:** Balance, Older adults, Compensatory stepping, Falls, The Microsoft Kinect™

## Abstract

**Background:**

Rapid compensatory stepping plays an important role in preventing falls when balance is lost; however, these responses cannot be accurately quantified in the clinic. The Microsoft Kinect™ system provides real-time anatomical landmark position data in three dimensions (3D), which may bridge this gap.

**Methods:**

Compensatory stepping reactions were evoked in 8 young adults by a sudden platform horizontal motion on which the subject stood or walked on a treadmill. The movements were recorded with both a 3D-APAS motion capture and Microsoft Kinect™ systems. The outcome measures consisted of compensatory step times (milliseconds) and length (centimeters). The average values of two standing and walking trials for Microsoft Kinect™ and the 3D-APAS systems were compared using *t*-test, Pearson’s correlation, Altman-bland plots, and the average difference of root mean square error (RMSE) of joint position.

**Results:**

The Microsoft Kinect™ had high correlations for the compensatory step times (*r* = 0.75–0.78, *p* = 0.04) during standing and moderate correlations for walking (*r* = 0.53–0.63, *p* = 0.05). The step length, however had a very high correlations for both standing and walking (*r* > 0.97, *p* = 0.01). The RMSE showed acceptable differences during the perturbation trials with smallest relative error in anterior-posterior direction (2-3%) and the highest in the vertical direction (11–13%). No systematic bias were evident in the Bland and Altman graphs.

**Conclusions:**

The Microsoft Kinect™ system provides comparable data to a video-based 3D motion analysis system when assessing step length and less accurate but still clinically acceptable for step times during balance recovery when balance is lost and fall is initiated.

## Background

The ability to perform rapid compensatory balance reactions (i.e., stepping movement) when balance is lost has been linked to fall risk in older adults [1–3]; however, the assessment of these reactions is commonly undertaken in a laboratory. Measurement tools for assessing compensatory balance reactions include force platforms, electromyography systems, as well as full-body three dimensional (3D) kinematic assessments [1–4]. The examination of compensatory stepping behavior utilizes expensive motion analysis equipment to analyze those balance reactions that would not be available to most elderly individuals or even rehabilitation clinics.

With regard to clinic-based assessments, there are simple objective clinical measures of balance control, such as Berg Balance Scale, Timed get up and go, and Short Physical Performance Battery. These measures include clinician assessments of quality of movement, and timing using a stopwatch. While providing useful information to the clinician, they are prone to ceiling effects and often cannot accurately quantify the postural control strategies being used by the patient; they are usually designed for frail older adults with severe deficits in their postural control system. Furthermore, these measures cannot provide information regarding a subject's ability to prevent falling if balance is lost. Adding more advanced data collection and analysis tools such as force platforms, electromyography, and 3D motion analysis systems allows analysis of balance recovery reaction in finer detail. For example, a 3D kinematic video-based motion analysis system can be incorporated into the testing protocol to measure spatiotemporal factors such as trajectories and timing of rapid leg movement during compensatory stepping, which have been shown to discriminate between young and older populations as well as between frail and healthy older adults [1–3]. This is most commonly achieved using systems that require multiple cameras and tracking markers placed on the skin, making them cumbersome and expensive, and requiring extensive technical expertise to operate and interpret. This combination of factors precludes their use in all but the major clinical centers and research laboratories. However, a recent development in computer gaming technology – the Microsoft Kinect™ system – is inexpensive, portable, and does not require markers to determine anatomical landmarks; consequently it may overcome the limitations associated with laboratory-based movement analysis systems.

The Microsoft Kinect system incorporates infra-red light and a video camera to create a 3D map of the area in front of it [5], and uses a randomized decision forest algorithm to automatically determine anatomical landmarks on the body, such as joint centers, in close to real time [6]. The results of previous studies are promising, and have shown that the depth sensor itself is accurate for assessing 3D position in a workplace environment [7], and that joint centers derived from the Microsoft Kinect system can be used to classify dance gestures [8]. If the positions of these reported anatomical landmarks are found to be accurate during the assessment of compensatory stepping reactions, which are very rapid, this could facilitate advanced analysis of these recovery reactions to be performed in the clinical setting.

Compensatory stepping reactions have long been considered an important focus of research especially in older persons. External perturbations of posture, such as a slip or a trip, trigger automatic compensatory postural responses that act to recover equilibrium with a delay of about 100 ms. The recovery responses are specific to the size, type, and direction of the imposed perturbation [2, 9–11]. If the person cannot regain balance, a compensatory step will be initiated [2, 12]. The compensatory step strategy is the most important postural response that can directly prevent a fall [1–3, 13]. These types of responses have been studied extensively in healthy human participants [14–17] as well as in older participants [18–20] and in different patient populations [21–23]. Surprisingly, these postural “reflexes” were less studied during walking although most falls occurred during locomotion (i.e., walking). Age-related deterioration in balance compensatory responses especially during walking may be a major contributor to falls in older adults. In fact, the inability to step rapidly in response to a loss of balance experienced in everyday life ultimately determines whether a fall occurs. Thus it is important to examine these compensatory responses in geriatric and rehabilitation clinics and measure whether these skills can be improved as a result of training. Today most rehabilitation clinics are unable to measure these important skills in part due to high cost and time demands.

The ability to differentiate postural control strategies using an inexpensive, portable, and widely available system could provide clinical and research benefits in examination and treatment of older adults as well as patient populations. Consequently, the aim of this study was to assess the concurrent validity of the anatomical landmarks collected using Microsoft Kinect with a kinematic assessment tool (i.e., a 3D computer-assisted video motion analysis, the Ariel Performance Analysis System) during standing and walking, as well as the compensatory step recovery reactions after loss of balance resulting from unexpected perturbation of posture during standing and walking.

## Methods

In an explorative laboratory study, 8 healthy young adults (21–29-years-old) were recruited from the university population. Participants were signed on an informed consent and approval by the Helsinki committee of Barzilai University medical center, Ashkelon, Israel (ClinicalTrials.gov Registration number #NCT01439451).

### Experimental protocol

In the first stage of the experiment participants were instructed to stand upright in a narrow base standing (heels and toes touching). In the second stage of the experiment, the participants were exposed to two unexpected right and left perturbations with maximal acceleration of 9.8 m/s^2^ and a top velocity of 0.7 m/s and 10 cm horizontal translation movement. Participants were instructed to respond in a "natural" manner (no instructional constraints). The participants had no knowledge regarding the direction and the timing of perturbation. In the third stage of the study we recorded the participants during comfortable treadmill walking. Finally, the participants were exposed to unexpected right and left platform perturbations (i.e., similar to the standing trails), while the participants walked on the treadmill [24]. Thus each subject went through 4 perturbations: two while standing, and two while walking. To prevent injury during loss of balance, the participants wore loose safety harnesses that arrested the fall, but allowed them to execute step recovery reactions.

Kinematic data during the experiment were collected with 3D Ariel Performance Analysis System (APAS) sampled at a frequency of 60 Hz and stored on a hard disk for later processing.

At the same time, data from the Microsoft Kinect system, sampled at a frequency of 30 Hz, was also stored on a hard disk for later processing. The Ariel Performance Analysis System is a computer-assisted video motion analysis evaluating human kinematics both in research and clinical applications. Klein and DeHaven [25] determined the upper limits of accuracy and consistency of linear and angular measures obtained using the Ariel Performance Analysis System. Reference standards included a meter stick and a universal 360° goniometer. Average mean error observed for reconstruction of absolute point estimates was found to be less than 3.5 mm. Mean error estimate for 3D reconstruction of a linear standard was found to be 1.4 mm (SD 0.30). Average mean angular error observed for 3D reconstruction of goniometer settings of 10° to 170° was found to be 0.26° (mean SD 0.21). In this experiment we compared the different measurements that were recorded by the Ariel Performance Analysis System and Microsoft Kinect system. Recently, Clark et al. [26] found that the Microsoft Kinect can validly assess kinematic strategies of postural control (i.e., forward reach, lateral reach, and single-leg eyes-closed standing balance). The Accuracy of Kinect landmark movements against Vicon marker locations was found to be very high for upper extremity and trunk movements and lower for ankle and foot motion depended on movement dimension, landmark location and performed task [27]. In general vertical movements had the lowest correlations between both systems.

### Data analysis

First, we used the following data transformations:

1. Each Kinect joint was matched with the appropriate APAS marker. The Kinect X-axis is switched with the APAS Z-axis (i.e., mediolateral direction) thus Kinect measures were multiplied by −1.
2. The APAS system measures distance in centimeters, thus the Kinect measurements were translated to centimeters.
3. The two systems have a different absolute zero. To normalize both systems we used the average position of the first 10 recorded seconds of the experiment, when the participants were standing still, as the absolute 0 position for each system.
4. We smoothed the Kinect readings using a simple first order filter with *k* = 5. That is, the smoothed value of a Kinect data point at time *t* is the average of the raw data points from *t-2* to *t + 2*.
5. The two systems, APAS and Kinect, did not start recording at the exact same time. Hence, we had to compute a time offset to match the readings from the two systems on the time axis. To do so, we used only the extreme data points of the left ankle X-axis (i.e., mediolateral direction). The APAS system able to "identify" accurately the platform horizontal translation movement since marker was placed on the perturbation system. However the Kinect system is not designed to detect perturbation system position. Note that this is not a true limitation, as the perturbation system is controlled by a computer that was connected to the Kinect sensor. Thus, it was simple to record the Kinect readings on the same time scale as the perturbation system control signals.

Following the data transformations we computed the root of the mean square error (RMSE) of the Kinect measurements compared with the APAS measurements. For each joint j, the error for a time point t for the Kinect measurement is computed using

$$ Erro{r}_t= Kinec{t}_t- A P A{S}_{t\hbox{'}} $$

where *APAS*_*t’*_ is the APAS reading at the time point closest to *t*, which is always within 1 millisecond of *t*. Then, the RMSE of the Kinect system with respect to the APAS measurements is computed using

$$ RMSE=\sqrt{\frac{\sum_t Erro{r}_t^2}{n}} $$

Two parameters were measured: 1) the length of the compensatory stepping response (i.e., step length); 2) The step reaction time and step execution duration. The following events were extracted from the motion analysis APAS system and the Microsoft Kinect system: (1) The unexpected platform perturbation was detected as the first medio-lateral deviation of the perturbation system and from the computer that was connected to the Kinect sensor respectively; (2) Foot-off was defined at the sudden elevation of the foot off the ground using the ankles’ vertical values; (3) Foot-contact was defined as the foot contacted the ground using the ankles’ vertical values; (4) Compensatory step initiation time (in milliseconds) was calculated as the time from perturbation to foot-off the ground; (5) Compensatory step duration (in milliseconds) was calculated as the time from platform perturbation to foot contact the ground; (6) Compensatory step length (cm) was calculated as the displacement of the ankle marker from the beginning of the step to the end of the step in mediolateral direction.

### Statistical analysis

Independent *t*-test for independent measures were used to evaluate the differences between APAS and Microsoft Kinect systems to measure step parameters during the standing and walking trials (*p* < 0.05). Once the timing of each temporal event was determined, the average values of the two perturbation trials standing as well as walking for each outcome measure for the Microsoft Kinect and 3D APAS methods were compared using Pearson’s *r* correlation, ordinary least products (OLP) regression [28]. The following guidelines were used when interpreting Pearson’s *r* magnitudes: absent to little (*r* = 0.00–0.25), low (0.26–0.49), moderate (0.50–0.69), high (0.70–0.89), or very high (0.90–1.00) [29]. All statistics were analyzed using SPSS (version 16, Chicago, IL USA).

Additionally, to assess systematic changes of the mean, Bland and Altman analyses were used [30], including the following calculations:

1. d = mean difference between the two measurement methods.
2. SDdif = SD of the difference between the two measurement methods.
3. 95% confidence interval of d (95% CI).
4. Limits of Agreement (LOA) = d ± 1.96 SDdif.

Graphics were used to improve analyses of Kinect and APAS systems from the four different trials and to guide interpretation of discordance patterns. This included plotting a difference in mean from "Bland and Altman plots" [30]. The Bland and Altman graphs depict the measurement error as the differences (y-axis/vertical direction) between Kinect system and APAS plotted against the mean (x-axis/mediolateral direction) of the two trails for each subject. The advantage of Bland and Altman plots is in examining the differences for each parameter measured by the two systems. The plots also provide LOA, when most differences (95%) lie in LOA, normal distribution of differences can be assumed. Bland and Altman plots were generated using MedCalc (version 14.10.2.0, MedCalc Software bvba, Ostend, Belgium).

## Results

A total of 32 compensatory stepping trials from 8 participants were assessed and analyzed by the APAS and Microsoft Kinect systems.

**Fig. 1:**
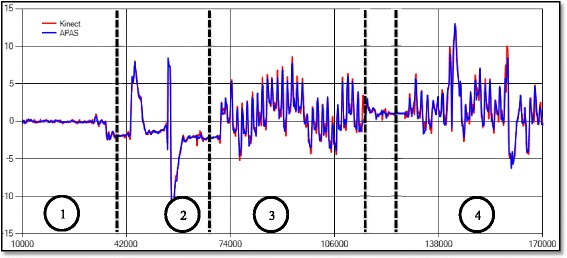
Example of kinematic analysis using motion analysis system (APAS Inc.) vs. Microsoft Kinect system, captured locations of marker placed on the left ankle, x-axis /mediolateral direction in a single subject. The x-axis /mediolateral direction shows time (in milliseconds), and the y-axis/vertical direction shows the position (in cm). The circled numbers denote the phases of the experiment: 1) standing; 2) perturbations while standing; 3) walking; 4) perturbations during walking

**Fig. 2:**
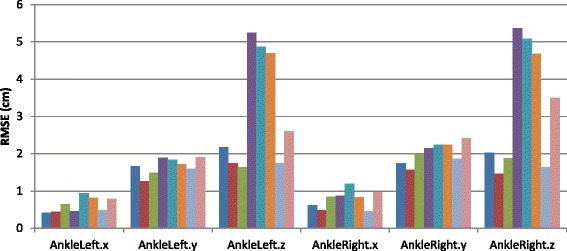
RMSE in cm over the ankles throughout the experiment for all participants. Note: the RMSE is almost always less than 1 cm for the x-axis /mediolateral direction and less than 2 cm for the y-axis/vertical direction. The z-axis/anterior-posterior direction errors are larger, and for some participants the errors exceed 5 cm

Regarding the RMSE of the Microsoft Kinect system with respect to the APAS system, Fig. 1 show the locations of marker placed on the left ankle in all phases of the experiment. Figure 2 shows the RMSE (in cm) for the ankles over all axes; the RMSE is almost always less than 1 cm for the X-axis (mediolateral direction), and less than 2 cm for the Y-axis vertical direction). The RMSE for the Z-axis (anterior-posterior direction) are larger, and for some participants the errors exceed 5 cm.

Regarding the RMSE in cm of the Microsoft Kinect system with respect to the APAS system for hip, shoulder, and wrist joints markers, Tables 1 and 2 show that these joints move less than the ankle joint during comfortable treadmill walking, and thus the recorded values and the differences between the systems are smaller. Still, in all joints the accuracy over the Z-axis (anterior-posterior direction) is considerably lower than the accuracy over all other axes, however since ankle movements in the vertical plane (y-axis) was very relatively small during the trails, the relative RMSE in % was the largest.

**Table 1:** Average difference (RMSE) of joint position between APAS and Kinect systems measured in cm. Note: the z-axis is the anterior-posterior displacement; the y-axis is the vertical displacement; and the x-axis is mediolateral displacement

	Hip						Shoulder						Wrist					
	Left			Right			Left			Right			Left			Right		
	x	y	z	x	y	z	x	y	z	x	y	z	x	y	z	x	y	z
1	0.53	0.55	1.54	0.55	0.61	1.88	0.79	0.68	1.86	0.76	0.44	1.82	0.84	0.85	1.79	0.63	0.73	1.80
2	0.54	0.85	2.30	0.56	0.96	2.88	0.63	0.63	1.22	0.64	0.45	0.81	0.52	0.73	1.19	0.42	0.72	0.97
3	0.67	0.60	1.47	0.56	0.91	1.43	0.79	0.53	1.53	0.68	0.43	0.82	0.61	0.68	1.06	0.60	0.81	1.36
4	0.52	0.73	2.47	0.56	0.64	2.24	0.49	0.65	3.11	0.40	0.43	1.35	0.48	0.53	1.38	0.65	0.67	1.31
5	0.85	0.79	2.20	0.82	0.56	1.18	0.29	0.57	0.61	0.41	0.78	2.34	0.89	1.58	3.01	0.84	1.60	2.54
6	0.94	0.71	1.02	0.85	0.83	2.30	1.43	0.89	1.70	1.29	0.63	1.80	1.27	1.40	2.22	1.10	1.21	1.78
7	0.43	0.55	0.89	0.45	0.55	0.83	0.57	0.51	0.53	0.47	0.51	0.58	0.45	1.11	0.77	0.47	0.88	0.89
8	0.65	1.13	2.72	0.57	0.79	2.64	0.46	0.49	1.24	0.46	0.49	1.24	0.54	0.72	1.50	0.49	0.79	1.35

**Table 2:** Relative errors measured in cm

	Left ankle			Right ankle		
	X	Y	Z	X	Y	Z
Standing	0.14 (29%)	0.49 (38%)	0.4 (16%)	0.13 (29%)	0.39 (37%)	0.4 (17%)
Standing + Perturbation	0.46 (2%)	0.61 (13%)	0.75 (7%)	0.55 (2%)	0.88 (11%)	1.08 (7%)
Walking	0.68 (7%)	1.99 (16%)	4.08 (6%)	0.72 (7%)	2.15 (16%)	3.97 (6%)
Walking + Perturbation	0.8 (3%)	2.29 (13%)	4.4 (6%)	1.05 (3%)	2.5 (13%)	4.31 (6%)

The RMSE (averaged over all participants) and the relative RMSE (relative to the maximal movement – difference between max and min). Note: the z-axis is the anterior-posterior displacement; the y-axis is the vertical displacement; and the x-axis is mediolateral displacement

**Fig. 3:**
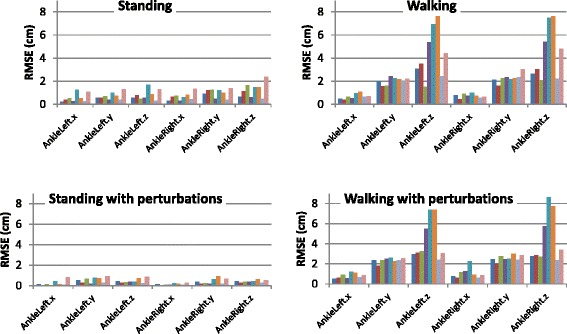
RMSE (in cm) of the ankle in the various phases of the experiment. The RMSE while standing is relatively low compared to the RMSE while walking. Perturbations do not significantly affect the RMSE

We further analyzed the RMSE in the different phases of the experiment 1) standing; 2) perturbations while standing; 3) walking; and 4) perturbations while walking. Figure 3 compares the RMSE in the various phases. As expected, the RMSE while standing is relatively low compared to the RMSE while walking. Perturbations do not significantly affect the RMSE, compare with the standing and walking trials (Table 2).

**Fig. 4:**
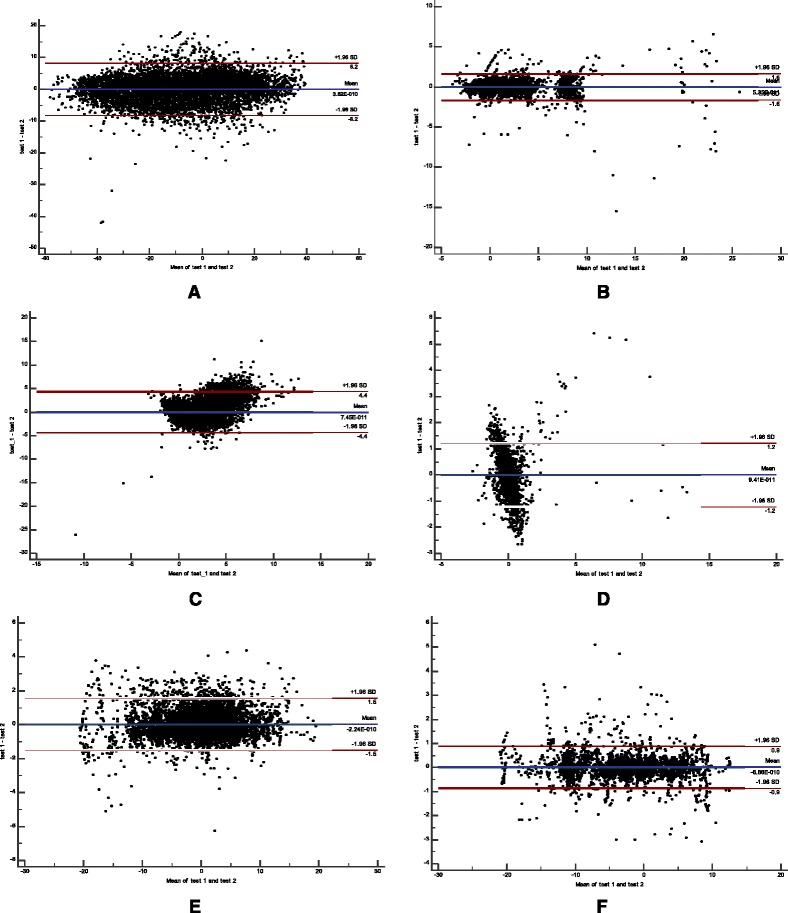
Bland and Altman graphs for left ankle location for 8 participants in perturbation during walking in (**a**) the z-axis/anterior-posterior displacement; (**c**) the y-axis/vertical displacement; and (**e**) the x-axis /mediolateral displacement, and during standing in: (**b**) the z-axis/anterior-posterior displacement; (**d**) the y-axis/vertical displacement; and (**f**) the x-axis/mediolateral displacement. The difference (anterior-posterior, vertical and mediolateral displacements) between the motion analysis (APAS Inc.) vs. Microsoft Kinect systems is plotted against the mean for each subject. Mean difference between the two tests: *solid line*; 95% confidence interval of the mean difference: *dotted line* (95% CI); limits of agreement: *dashed line* (LOA)

**Fig. 5:**
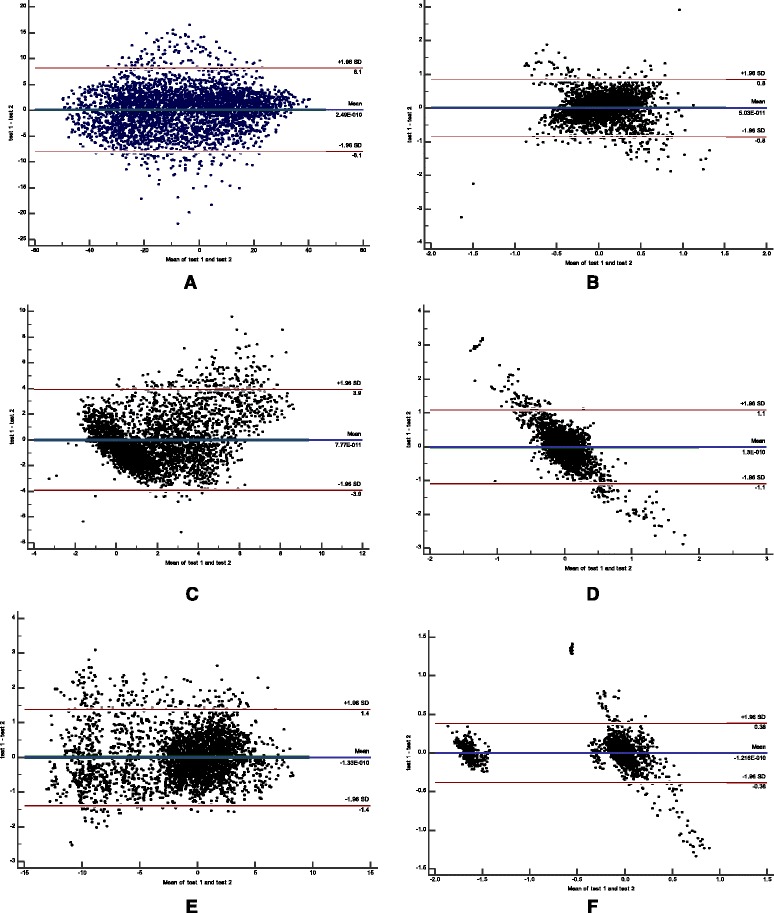
Bland and Altman graphs for left ankle location for 8 participants during walking in: (**a**) the z-axis/anterior-posterior displacement; (**c**) the y-axis/vertical displacement; and (**e**) the x-axis /mediolateral displacement, and during standing in: (**b**) the z-axis/anterior-posterior displacement; (**d**) the y-axis/vertical displacement; and (**f**) the x-axis/mediolateral displacement. The difference (in anterior-posterior, vertical and mediolateral displacements) between the motion analysis (APAS Inc.) vs. Microsoft Kinect systems is plotted against the mean for each subject. Mean difference between the two tests: *solid line*; 95% confidence interval of the mean difference: *dotted line* (95%CI); limits of agreement: *dashed line* (LOA)

In addition, we found no systematic changes over time were evident in the Bland and Altman graphs (Figs. 4 and 5), i.e. zero was included in the 95% confidence interval of d and most differences (95%) of all values of the left ankle location in all 3 planes were within LOA.

The temporal and special measures of compensatory step are presented in Table 2. There were no statistically significant differences between Microsoft Kinect and APAS systems across all compensatory step execution parameters for both standing and walking conditions with the exception of the compensatory step time (ms) in the left leg during walking trials. The compensatory step time (ms) measured by the Microsoft Kinect system was significantly slower than compensatory step time (ms) measured by the APAS system (Table 3).

**Table 3:** Mean of step execution parameters for young participants

A. Compensatory step during standing, mean(SEM)		Kinect	APAS	Difference
Right	Compensatory Step time (ms)	425.1 (69.3)	397.2 (84.3)	28
	Step length(cm)	17.6 (3.8)	17.9 (3.7)	0.3
Left	Compensatory Step time (ms)	271.5 (27.2)	251.0 (29.4)	20
	Step length(cm)	20.2 (4.9)	18.9 (4.8)	1.3
B. Compensatory step during walking, mean(SEM)		Kinect	APAS	
Right	Compensatory Step time (ms)	309.3 (25.5)	376.1 (59.4)	66.9
	Step length (cm)	19.2 (3.2)	19.9 (3.1)	0.7
Left	Compensatory Step time (ms)	635.5 (78.5) *	476.2 (46)	158.8
	Step length (cm)	12.3 (3.4)	13.2 (3.8)	0.9

*Abbreviations*: *cm* centimeters, *ms* milliseconds

Values shown represent average±1 standard deviation in milliseconds (ms) for step times and centimeters (cm) for step length

^*^ Indicates statistically significant differences between the APAS and Kinect systems using *T*-test (*p* < 0.05)

We also analyzed the correlations for the compensatory step times between APAS and Microsoft Kinect systems (Table 4). During the perturbation standing trials the correlations were excellent for compensatory step times (*r* = 0.754–0.789) and for the compensatory step length (*r* = 0.977–0.982). During the perturbation walking trials, however, the correlation is fair (*r* = 0.533–0.632) for compensatory step times but excellent for compensatory step length (*r* = 0.993–0.994).

**Table 4:** Pearson’s r correlation between the Microsoft Kinect™ and 3D APAS methods for compensatory stepping time and length. The average values of the two perturbation trials in standing and walking trials

		Compensatory Step time (ms)	Step length (cm)
Standing	Left leg	0.789 (*p* = 0.04)	0.977 (*p* = 0.01)
	Right leg	0.754 (*p* = 0.04)	0.982 (*p* = 0.01)
Walking	Left leg	0.632 (*p* = 0.05)	0.993 (*p* = 0.01)
	Right leg	0.533 (*p* = 0.05)	0.994 (*p* = 0.01)

*Abbreviations*: *cm* centimeters, *ms* milliseconds

## Discussion

In the present study, we evaluated the suitability of the Microsoft Kinect system for clinical use for evaluation of the timing and displacement data of the foot during execution of rapid compensatory stepping against a reference system, namely APAS. We analyzed landmark movement accuracies as well as the accuracy and reliability of different clinical parameters derived from 4 motor tasks (i.e., standing, perturbations while standing, walking, and perturbations while walking.) in young healthy subjects. The results can only be generalized for young adults thus should be taken in caution. In general our results suggest that there were differences in signal accuracy according to 1) directional components (highest for x axis/the mediolateral displacement), 2) landmark location (lowest for feet due to a large feet displacement) and 3) performed movement task (highest for standing). The last is possibly attributable to the differences in movement amplitudes. As one conclusion of this study, 3D mediolateral positions of the feet can validly be used for step execution movement analyses during standing and calculation of clinical parameters.

Concerning the differences in accuracy between the directional components, our data show highest accuracy ML movements, while other reports [27, 31] found highest accuracy in AP movements. One explanation is that the accuracy increases with larger movements as larger signals improving the accuracy. In our experiment participants walked on a treadmill thus their step length in AP direction was rather smaller than over ground walking (15-20 cm vs with 75 cm, respectively) also a difficulty to locate both the initial contact of the foot with the ground and the foot off the ground might reduce accuracy in AP direction. However, the onsets of both the right or left horizontal platform perturbations and the mediolateral movement compensatory of the foot was easy to detect and the range of mediolateral displacement of the foot was rather large (15-20 cm) compared with a very small ML feet displacement in just walking. This is supported by Clark et al. [31] who suggested that the accuracy of movement signals is influenced by larger range of motion since the noise is proportionally smaller in signals with larger range. This is supported by our findings, we found high accuracy for ML in treadmills perturbation experiments in both during walking, and standing where the relative errors were small (2–3%) compare with 29% during standing and 7% in walking where there were a very small range of feet motion in the ML direction. This is supported by the relative RMSE values in vertical direction which were the smallest for feet movements, since the vertical component of feet displacement was the smallest (around 5 cm). Thus in terms of noise behavior, the instability of landmark locations according to RMSE reflected in general the lower signal agreement for the same landmarks. For clinical application of measurements of compensatory step, we therefore recommend to examine ability to execute stepping of a large perturbations which trigger large step execution movements. For landmarks with a lower accuracy such as movements in the AP direction, different recording angles may be explored to increase accuracy, also advanced filtering techniques or alternative skeleton models may also derive more accurate clinical parameters even for small movements, like step recovery reactions to a smaller perturbations magnitude in ML as well as for AP directions.

Our study was focused on the ability of the Kinect system to measure accurately clinical parameters that reflect the ability to recover from a sudden loss of balance i.e., the compensatory step responses. The clinical parameters of compensatory stepping where step execution time and step length, are well recognized as the most important parameters that can prevent from falling when balance is lost unexpectedly. In our study the clinical parameters reflect foot displacement in the ML direction. These parameters showed high absolute agreement and no systematic bias between systems (see Bland and Altman graphs, Figs. 4 and 5), high consistency agreement and moderate absolute agreement. This may leads to assume that the Kinect system is accurate enough to measure clinical parameters of recovery stepping behavior especially in the mediolateral directions (i.e., step length and time). We also Table 3 show high Pearson's correlation coefficients against the APAS motion analysis for the lateral step length during the standing trials (*r* = 0.977–0.982) and during the walking trials (*r* = 0.993–0.994) and somewhat lower correlation coefficients for compensatory step times during standing trails (0.754–0.789) and the walking trials (0.533–0.632), this represent a lower ability to detect the onset of stepping while walking where to foot is in motion compare with higher ability to detect step onset during the standing trials where the feet is stable prior the perturbations. There are several factors that might cause the results, first the Microsoft Kinect System has a relatively low temporal resolution (30 Hz), this may be insufficient to examine the compensatory step times during standing and walking where the limbs are rapidly accelerated post perturbation. Second, the Kinect may not be able to precisely detect the exact time when the perturbation system initiated the horizontal translation movement. In contrast, the 3D camera system was able to do so, since one reflective marker was placed on the moving platform and the system was able to detect this movement. Thus it is important to implement an automated identification mechanism that will be more robust and would allow to detect the platform movement initiation. Our analysis raveled also a very small differences in step length between the Kinect and APAS (0.3–1.3 cm) which is 1–6% from their step length, since the reliability of step length was not investigated and smallest detectable difference (SRD) was not evaluated it seems that this difference are not clinically different. Regarding the step times we found higher differences between Kinect and APAS but still small (6%–18%) and seem to be within the SRD, apart of the compensatory right step while walking (about 30% difference).

Since this is the first study to explore the ability of Kinect to accurately measure compensatory step responses we compare our results to a previous reports that show also high agreement with on gait analysis parameters generated from Vicon motion analysis system with Kinect [27, 31, 32] with respect to comfortable and maximum speeds including high accuracy and repeatability for step parameters during walking.

In summary, given that a number of previous studies have shown a link between compensatory stepping behavior and falls risk [1–3, 13], our results suggest that Kinect has the potential to be used in clinical screening programs as well as for training for an older adults at risk for falls. It seems that the dependency of movement signal accuracy on step length may impact derived clinical parameters of compensatory stepping. For instance, smaller movement show larger errors and are therefore more difficult to interpret. Our stepping data were derived from young's thus may be difficult to generalize to an older adults. However, older adults present a higher movement amplitude step length in a smaller perturbation levels since they are unable to recover their balance without stepping in a relatively small perturbations, this may positively affect signal accuracy with Kinect especially for "noisy" landmarks such as the feet. This may improve accuracy compared to our data in young participants. Thus we suggest to test this hypothesis on an older populations for a clinical application, repeatability measures may even prove better in older adults or other patient groups with more diverse motor performance. The results presented here help to select compensatory step parameter to be used in clinical setting in older adults and patient groups. While gait parameters such as walking speed are in use as a clinical measures, the current study show that compensatory step length especially in the ML direction that derived using the Kinect technology can be a reliable measure in clinical setting. The step time showed lower repeatability. The major benefits of Kinect for a clinical use is the cost, portability, and widespread availability of the system. An additional benefit is that the anatomical landmark data is automatically determined in close to real-time by the machine-learning algorithm that forms part of the Kinect system, and therefore the results can be provided to the patient almost immediately.

## Conclusions

The Microsoft Kinect system provides comparable data to a video-based 3D motion analysis system when assessing the compensatory step length in the mediolateral direction when balance is lost and fall is initiated. This could provide geriatric and rehabilitation practitioners with important information that is not currently obtainable in the clinical setting.

## Abbreviations

3D: Three dimensions
APAS: Ariel performance analysis system
Hz: Hertz
m/s: meters per seconds
m/s^2^: meters per seconds square
mm: millimeters
ms: milliseconds
OLP: Ordinary least products
RMSE: Root mean square error
SD: Standard deviation
cm: centimeters
SDK: Software development kit
